# fMRI indicates cortical activation through TRPV1 modulation during acute gouty attacks

**DOI:** 10.1038/s41598-019-48656-6

**Published:** 2019-08-26

**Authors:** Chiao-Chi Chen, Chen Chang, Yi-Hua Hsu, Yi-Jen Peng, Herng-Sheng Lee, Guo-Shu Huang

**Affiliations:** 1 Institute of Biomedical Sciences, Academia Sinica, Taipei, Taiwan; 2 Department of Pathology, Tri-Service General Hospital, National Defense Medical Center, Taipei, Taiwan; 3 Department of Pathology and Laboratory Medicine, Kaohsiung Veterans General Hospital, Kaohsiung, Taiwan; 4 Department of Radiology, Tri-Service General Hospital, National Defense Medical Center, Taipei, Taiwan; 5 Department of Medical Research, Tri-Service General Hospital, National Defense Medical Center, Taipei, Taiwan

**Keywords:** Sensory processing, Cortex, Experimental models of disease, Translational research, Rheumatoid arthritis

## Abstract

Gout is one of the most painful disease conditions. The central mechanism of pain processing in this condition remains elusive. Cerebral blood volume (CBV) responses are faithful correlates of brain activity changes; the application of CBV-weighted functional magnetic resonance imaging (fMRI) may shed light on the issue of interest. Transient receptor potential vanilloid 1 (TRPV1) is a critical ion channel expressed both peripherally in nociceptors and centrally in the brain. Whether TRPV1 plays a critical role in gout pain was also explored. Results showed that, in rats with gouty arthritis, noxious stimulation induced CBV increases in the primary somatosensory cortex and thalamus. These increases were correlated with up-regulated TRPV1 protein expression and pain behavior. Selective blockage of central TRPV1 channel activity by intrathecal administration of AMG9810 reversed the induced pain, and abolished the induced CBV increase in thalamocortical regions. The findings support that TRPV1 activation in the central pain pathway is crucial to the augmentation of pain in gouty conditions. This new information supports the development of TRPV1-based drugs for treating gout pain, while fMRI can be useful for repeated evaluation of brain activity changes induced by gout.

## Introduction

As one of the most painful disease conditions, gout is characterized by episodes of severe pain, tenderness, warmth, redness, swelling as well as fever^1^ in the affected tissue. However, there is very little information regarding the central mechanism underlying the experience of severe gout pain^2^. The spinothalamocortical pathway is one of the key pain circuits responsible for nociceptive processing in response to peripheral stimuli. The activation of this pathway upon pain stimulation can be detected *in vivo* by functional magnetic resonance imaging (fMRI) in both healthy humans and animals. Nevertheless, whether gout induces activity changes in this pathway or whether the alterations are responsible for the severity of the pain in gout has not been investigated.

Cerebral blood volume (CBV) weighted fMRI can be used in rodents. Its high signal-to-noise ratios are improved by the use of superparamagnetic iron oxide nanoparticles as the contrast agent^3–6^. CBV fluctuations highly correlate with brain activity changes. Therefore, CBV weighted fMRI is an alternative to the commonly used blood oxygen level dependent (BOLD) fMRI technique^7^; the quality of BOLD fMRI images tends to be poor because of low contrast to noise ratio and multiple hemodynamic factors^4^. The use of the CBV-weighted fMRI method in healthy rats has been shown to detect activity changes in the cortex and thalamus in response to noxious stimulation. Therefore its application is justified in the exploration of our issue of interest: how gout attacks affect the activity of the central nociceptive pathway.

To understand the gout-associated activity changes in the nociceptive signaling pathway, it is necessary to explore the underlying molecular mechanism. The significance of transient receptor potential vanilloid 1 (TRPV1) was of particular interest because it is a critical ion channel that responds to various physical and chemical stimuli including heat, pain, and capsaicin^8^. Activation of TRPV1 is linked to the painful conditions of inflammation^9–14^ and peripheral neuropathy^15^. Our hypothesis is that gout-induced change in nociceptive pathway activity may be mediated by an ion channel such as TRPV1, and results in exacerbation of pain.

In this study using CBV-weighted fMRI, we first examined whether normal and gouty conditions elicit different brain activity patterns in response to noxious electrical stimulation. TRPV1 protein levels were examined by western blotting and immunohistology from the neural tissues of the peripheral and central nociceptive pathways. The involvement of TRPV1 in gout pain was confirmed pharmacologically using a TRPV1-selective blocker called AMG9810. Altogether, these findings established the involvement of a TRPV1-mediated nociceptive mechanism in the augmentation of pain responses in gout. Such new information may support the development of TRPV1 antagonists as new drugs for treating gout, while fMRI can be used diagnostically for evaluating gout pain in the brain.

## Results

### The gouty arthritis rat model and pain behavior

**Figure 1:**
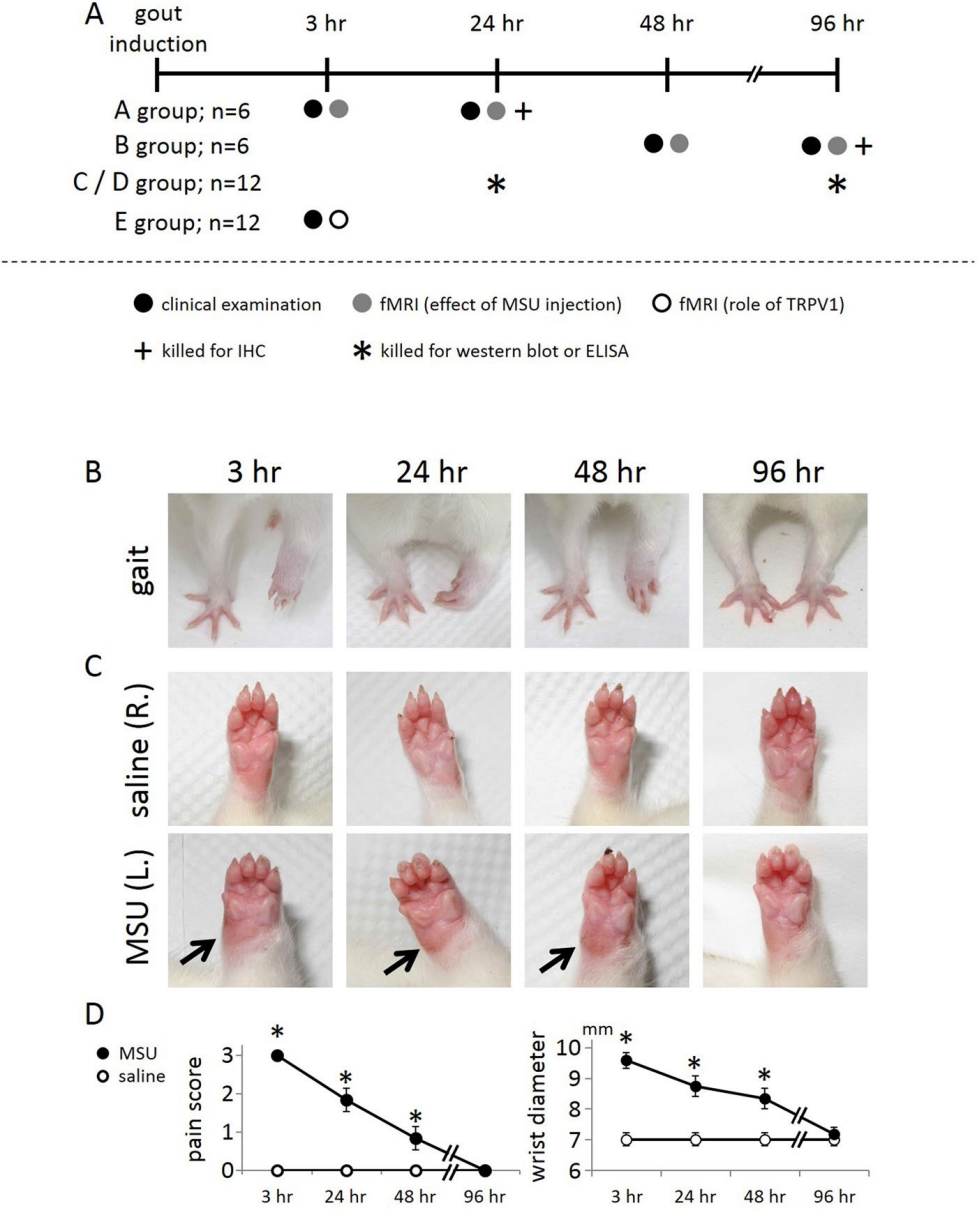
Overt signs in the wrist region of the rat with gouty arthritis with time. (**A**) Schematic showing the timeframe and experimental protocol. (**B**) Gait as an indicator of pain. (**C**) Swelling as a sign of inflammation. (**D**) Quantification of the gait and swelling over time indicates that gait abnormality and swelling resolved with time. Arrows point to the swollen spot. (*Indicates statistically significant difference with p < 0.05).

The left wrist was deposited with monosodium urate crystals (MSU) for the induction of gouty arthritis whereas the right wrist was treated with saline serving as the control. Signs of pain were observed at 3, 24, 48, and 96 hr after MSU deposition, as indicated in the timeline of Fig. 1A. Figure 1B shows that MSU treatment of the wrist resulted in an obvious gait abnormality when compared to the contralateral wrist. This gait change, indicative of the pain level, resolved with time. Figure 1C shows swelling of the MSU-treated wrist as compared to the contralateral wrist. This swelling sign also weakened over time. Pain scores and wrist diameters were statistically analyzed as shown in Fig. 1D, and the results of this analysis indicated that overt signs in this rat model of gouty arthritis were the most severe at 3 hr.

### CBV changes of the gouty arthritis rats

**Figure 2:**
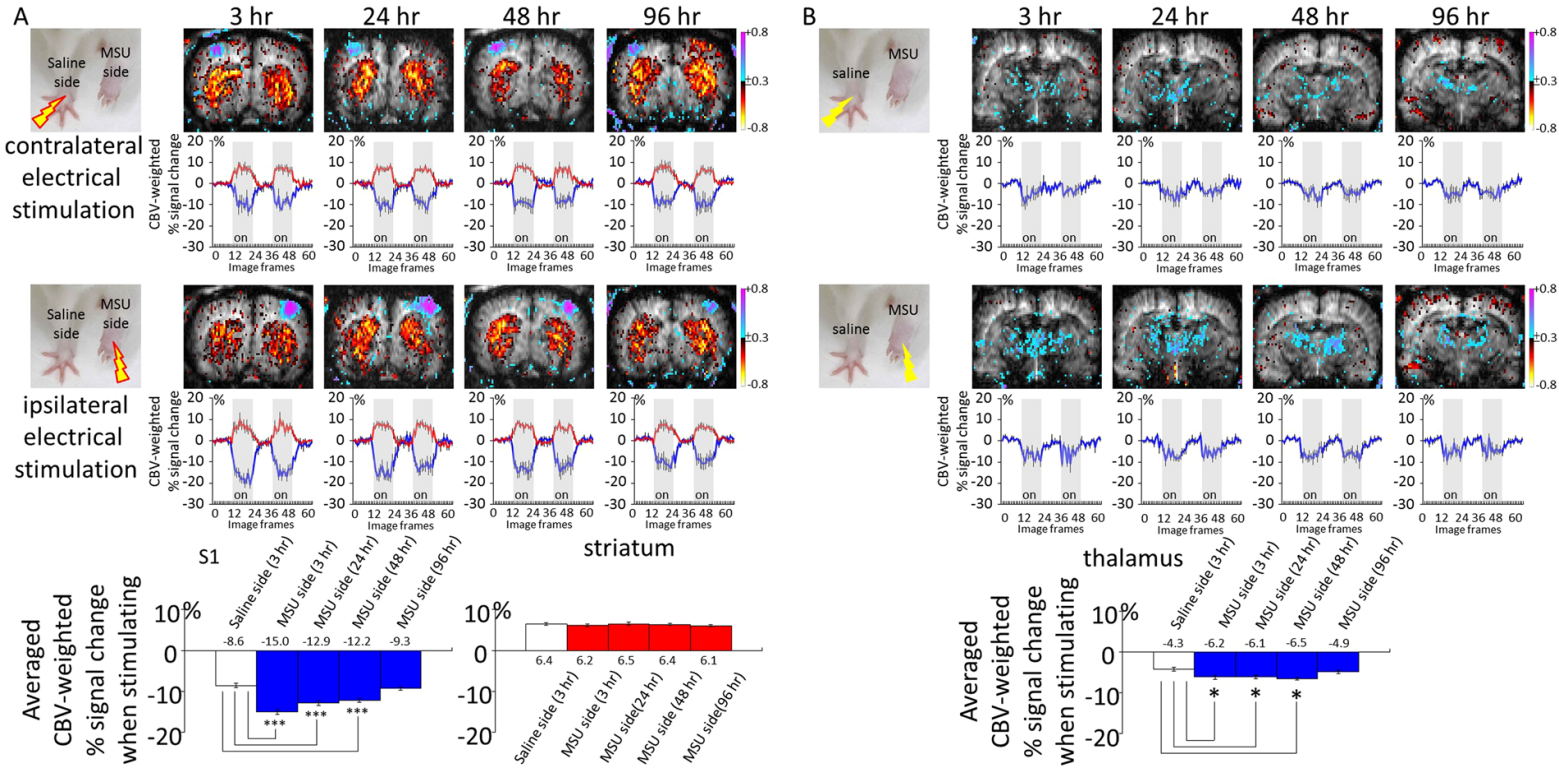
Pain evoked CBV-weighted fMRI was performed at 3, 24, 48, and, 96 hr after MSU deposition. (**A**) While stimulating the side of the forepaw injected at the wrist with only saline, a standard cortical and striatal CBV pattern was observed. Over time, this CBV pattern showed little change. On the other hand, when stimulating the side of forepaw injected at wrist level with MSU, much more pronounced cortical CBV responses were observed at the 3-hr time point and this response gradually weakened. The magnitude of the cortical response (blue) decreased with time. (**B**) As for CBV changes in the thalamus, stimulating the forepaw at the control side elicited diffuse CBV increases in the thalamus. In contrast, stimulating the forepaw on the MSU-treated side triggered more widespread CBV increases in the thalamus. This signal enhancement remained significant at 48 hr, yet was decayed at 96 hr. (* and *** indicate statistically significant differences with p < 0.05, and p < 0.001, respectively).

Evoked pain was assessed using CBV-weighted fMRI at 3, 24, 48, and, 96 hr after MSU deposition. CBV increases led to more accumulation of contrast agent in the region, and a drop in signal intensity (the blue curves in Fig. 2A,B). CBV decreases reduced the accumulation of regional contrast agent, and increased signal intensity (the red curves in Fig. 2A). As a result, negative signals changes are referred to as CBV increases and positive signal changes as CBV decreases.

While stimulating the side of the forepaw that was wrist-injected with only saline, a standard CBV pattern was observed on fMRI correlation maps as reported previously. That is, the cortex contralateral to the stimulated forepaw exhibited CBV increases whereas the bilateral striatum exhibited CBV decreases. The signal changes in the ROIs over the stimulation period are shown below the correlation maps and indicate that the cortex (blue) and the bilateral striatum (red) responded oppositely to the electrical stimulation. Over the different time points, this CBV pattern showed little change. On the other hand, when stimulating the side of the forepaw that was wrist-injected with MSU, a much more pronounced cortical CBV response (blue) was observed at 3 hr and weakened gradually over time. The bilateral striatal CBV changes, as a result of either saline or MSU injection, did not vary with time. Statistical analysis of the CBV changes indicates that the peak cortical response was reached at 3 hr after MSU deposition. This elevation remained significant at 48 hr but eventually decayed at 96 hr. Striatal CBV responses did not vary temporally.

CBV changes in the thalamus were also characterized, as shown in Fig. 2B. Stimulating the forepaw at the control side elicited diffuse CBV increases in the thalamus. These signal intensity changes were not very different over time. In contrast, stimulating the forepaw at the MSU-treated side triggered even more widespread CBV increases in the thalamus. This signal enhancement remained significant at 48 hr, yet was decayed at 96 hr.

### Peripheral inflammation and nociception of the gouty arthritis rats

**Figure 3:**
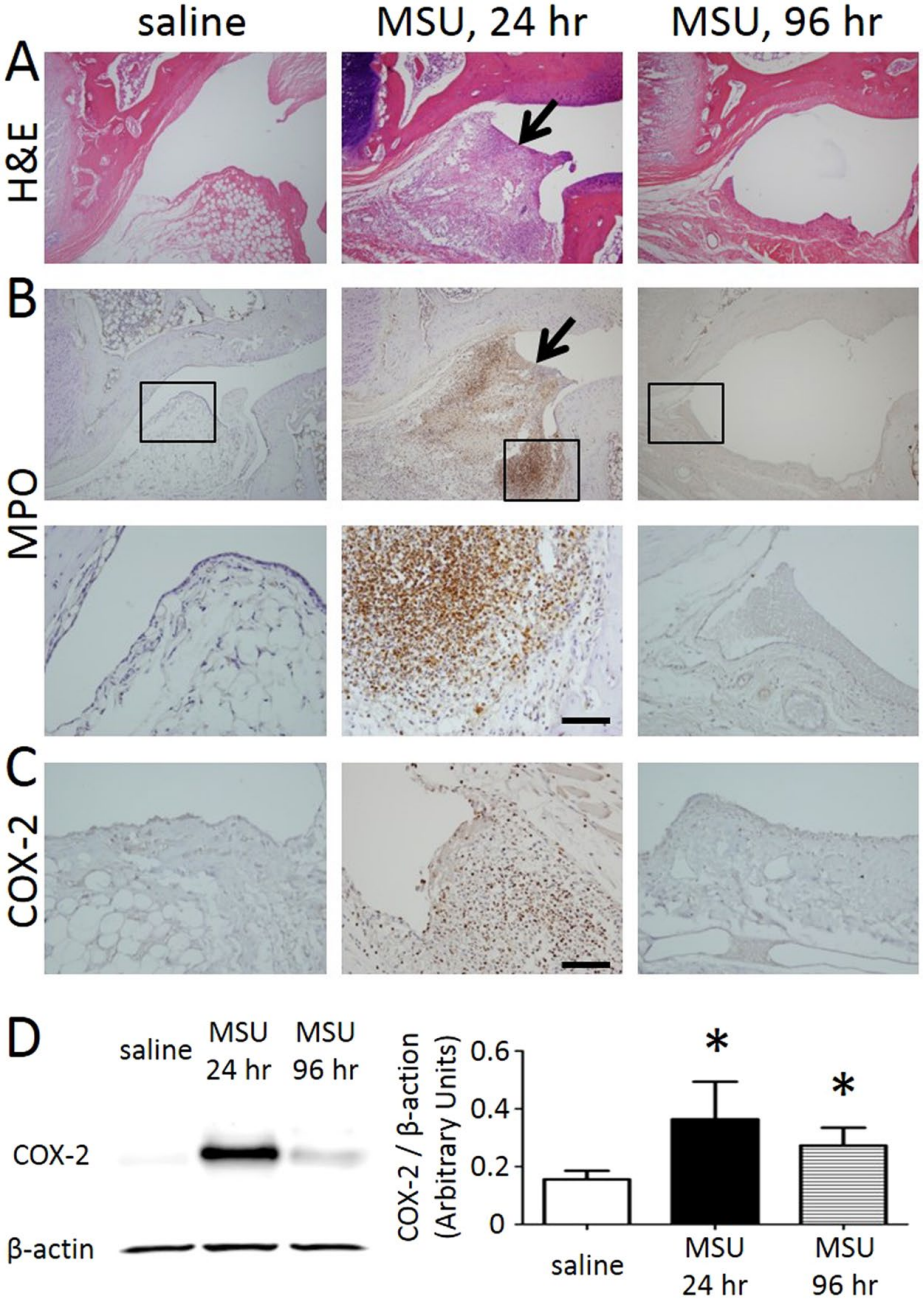
Inflammation of the wrist tissues examined by H&E staining (**A**, x40), MPO staining (**B**, x40), and COX-2 immunostaining (**C**, x200). The COX-2 expression was quantified by Western blot (**D**). The cropped images of the gels are shown in the figure, and images of the full-length gels are presented in Supplementary Fig. S1. (**A**) At 24 hr, the synovial membrane was infiltrated with numerous inflammatory cells, indicated by an arrow. These infiltrated cells were not observed in the saline-treated wrist tissue, and had obviously disappeared at 96 hr after MSU crystals injection. (**B**) The synovial tissues examined at 24 hours were filled with inflammatory cells (images shown at two different magnifications). (**C**) COX-2 immunostaining confirms the inflammatory nature of these infiltrated cells. (**D**) At 24 hr after MSU, COX-2 expression was at its highest level. Arrows point to enhanced staining of the wrist tissue. (* indicates statistically significant difference with p < 0.05 after ANOVA). Scale bar = 100 µm.

The wrist tissues harvested from the saline treated side or the MSU-treated side at 24 or 96 hr were examined histologically for inflammatory signs. H&E staining in Fig. 3A shows that, at 24 hr, the synovial membrane was infiltrated with numerous inflammatory cells, indicated by an arrow. These infiltrated cells were not observed in the saline-treated wrist tissue, and this site infiltration was obviously resolved at 96 hr after MSU crystals injection. Myeloperoxidase (MPO) staining in Fig. 3B confirmed that the synovial tissues examined at 24 hr were filled with inflammatory cells (images shown at two different magnifications). COX-2 immunostaining in Fig. 3C again confirms the inflammatory nature of these infiltrated cells. Quantitative analysis in Fig. 3D shows that at 24 hr after MSU, COX-2 expression measured as the signal intensity of COX-2 in relation to β-actin was at its highest level. The COX-2 levels decreased at 96 hr but remained significantly higher than that of the saline treated side.

**Figure 4:**
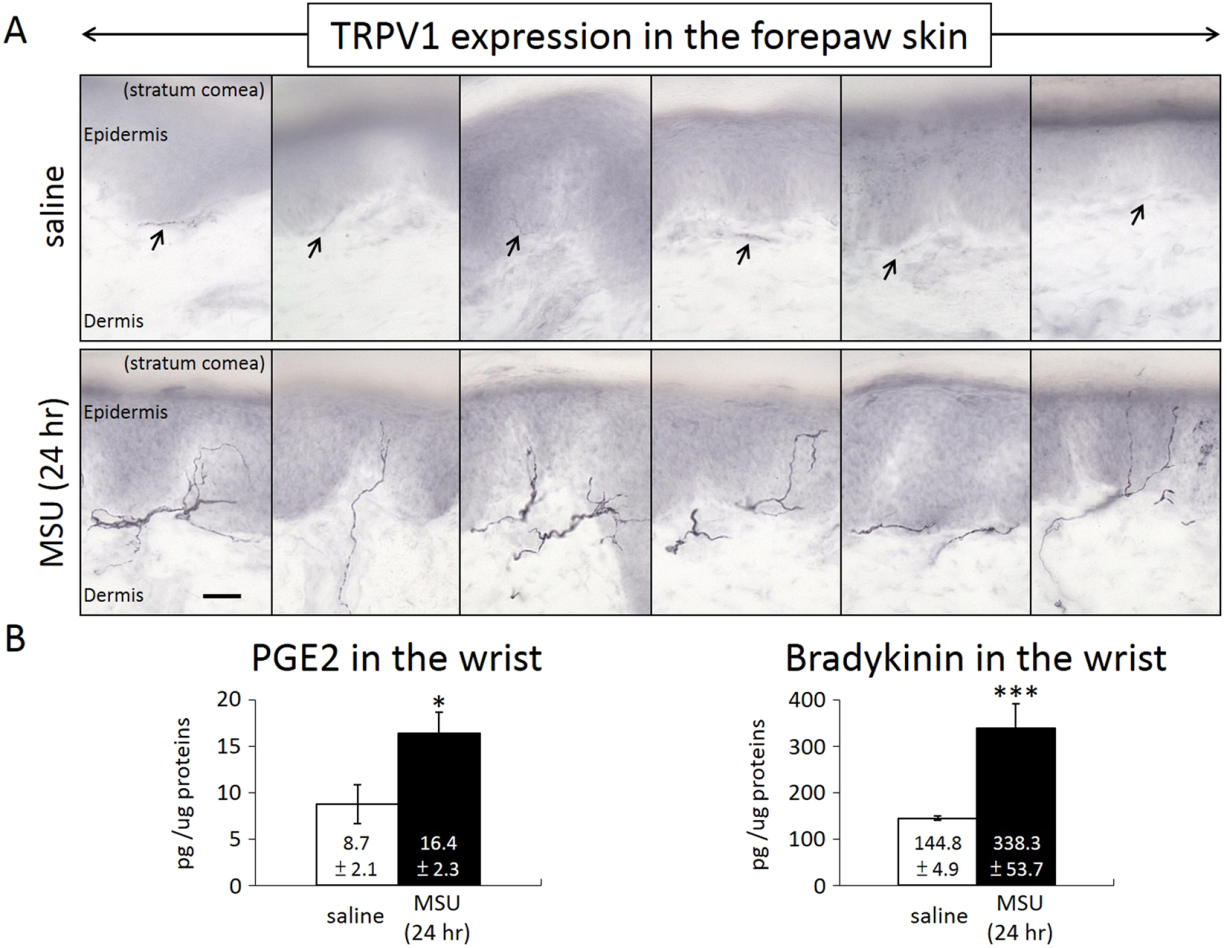
TRPV1 expression in the peripheral tissue and the levels of the TRPV1-associated mediators were assessed. (**A**) Distinct increases in TRPV1 expression were observed in the 24-hr MSU-treated wrist tissues, whereas very little TRPV1 signals were found in the saline-treated wrist tissues. Contiguous sections are shown. (**B**) PGE2 and bradykinin as TRPV1 mediators were both significantly up-regulated in the MSU-treated wrist regions. Arrows point to the scarcely stained nerve in the saline treated paw. (* and *** indicate statistically significant differences with p < 0.05, and p < 0.001, respectively, after ANOVA). Scale bar = 200 µm.

TRPV1 expression and TRPV1-associated mediators were examined in the peripheral wrist tissues. As shown in Fig. 4A, a distinct increase in TRPV1 expression was observed in the 24-hr MSU-treated wrist tissues, whereas very little TRPV1 signals were found in the saline-treated wrist tissues. ELISA assays in Fig. 4B showed that PGE2 and bradykinin levels as TRPV1 mediators were both significantly up-regulated in the MSU-treated wrist regions.

### Central TRPV1 expression of the gouty arthritis rats

**Figure 5:**
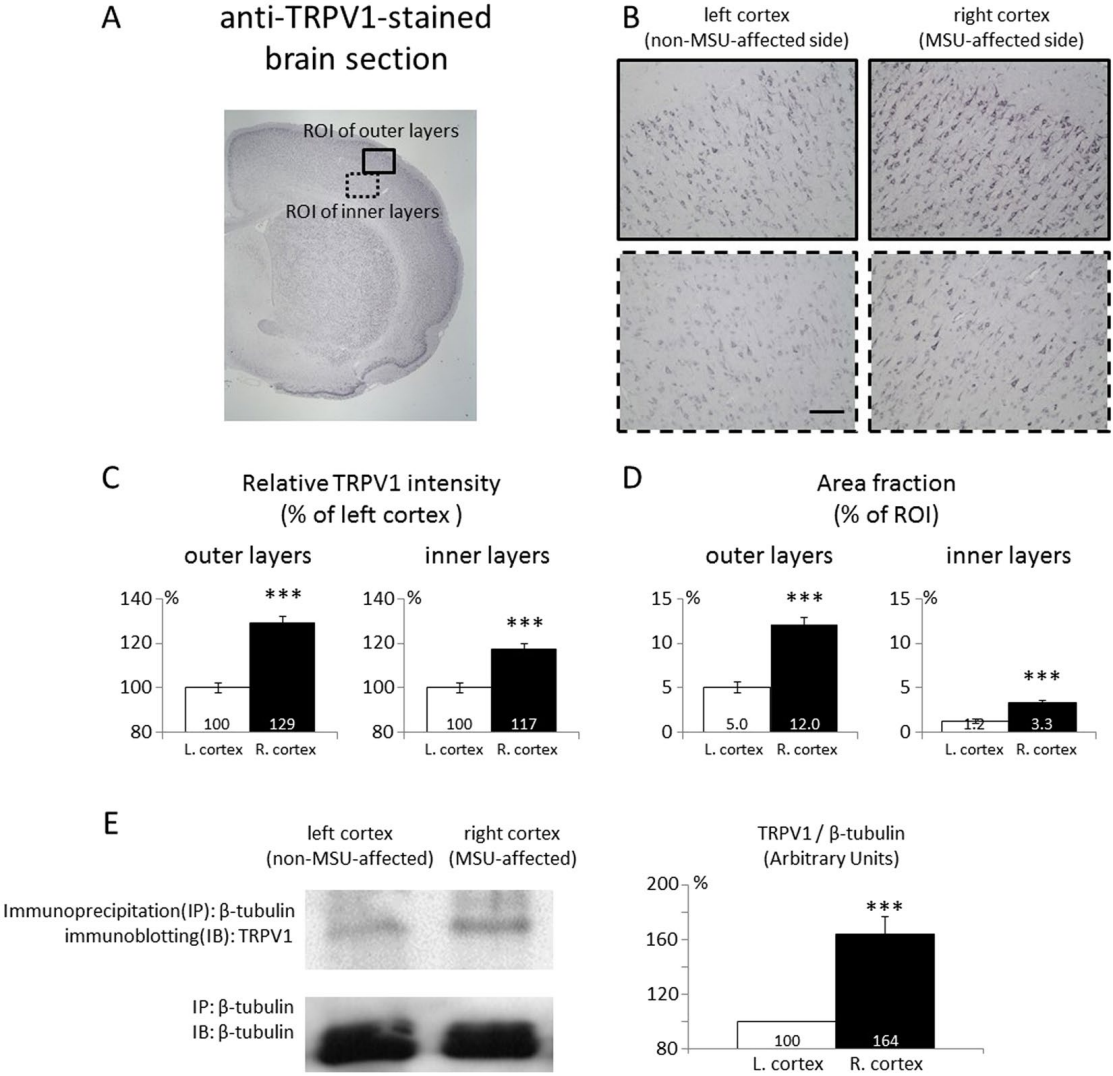
Cortical TRPV1 expression was enhanced by gouty arthritis. (**A**) ROI selection. (**B**) Cortical TRPV1 expression on the MSU-affected and control sides. (**C**) TRPV1 immunoreactivity intensity. (**D**) TRPV1 immunoreactivity coverage. (**E**) Immunoprecipitation of beta-tubulin with TRPV1 immunoblotting, which demonstrates a strong interaction between TRPV1 and neuronal filament proteins. The cropped images of the gels are used in the figure, and images of the full-length gels are presented in Supplementary Fig. S2. (***Indicates statistically significant difference with p < 0.001 after ANOVA). Scale bar = 50 µm.

TRPV1 expression was also examined in the cortical regions as indicated in Fig. 5A. Figure 5B shows that the TRPV1 expression of both the inner and outer layers of the cortex contralateral to the MSU treated side was significantly enhanced as compared to the contralateral cortex receiving signals from the saline treated side. The intensity and the coverage of TRPV1 immunoreactivity is summarized in Fig. 5C,D, respectively. Immunoprecipitation experiments indicate that TRPV1 binds significantly to β-tubulin in the affected cortical area, and thus supports the interaction between TRPV1 and neuronal structural proteins for modulating central nociceptive responses in gouty arthritis (Fig. 5E). Note that the immunoprecipitation experiments were carried out to assess the importance of TRPV1 with neuronal structures because TRPV1 western blotting alone did not reveal significant differences between the groups.

### The blocking effects of central TRPV1 expression in gouty arthritis rats

**Figure 6:**
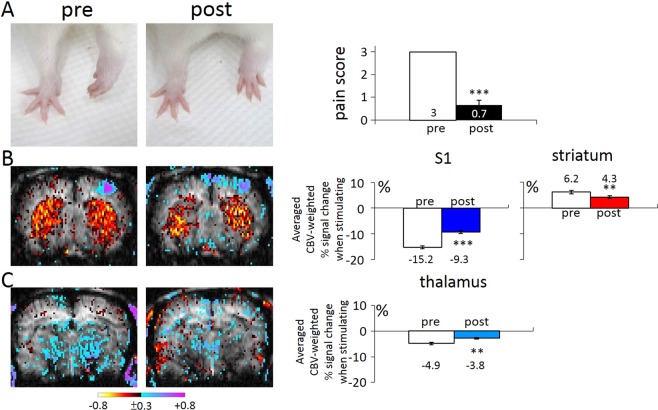
Intrathecal TRPV1 antagonism reversed the increase in gait score as a pain assessment, and the brain CBV responses in the cortical and thalamic areas. (**A**) The gait abnormality was reversed by the antagonist, with the average pain score decreasing from 3 to 0.7. (**B**,**C**) The cortical, striatal, and thalamic CBV responses were reduced significantly by the TRPV1 antagonist. (** and *** indicate statistically significant differences with p < 0.01, and p < 0.001, respectively, after ANOVA).

The pain reaction and fMRI responses were assessed before and after the infusion of the TRPV1 antagonist AMG9810. As shown in Fig. 6A, the gait abnormality was reversed by the antagonist, with average pain score decreasing from 3 to 0.7. The cortical, striatal, and thalamic CBV responses were reduced significantly by the TRPV1 antagonist. That is, AMG9810 mainly diminished the magnitude of the CBV increases or decreases, as shown in Fig. 6B,C. The results support the importance of central TRPV1 in modulating the pain of gouty arthritis.

## Discussion

The major interest of this study is to understand whether and how gout affects central nociceptive signaling using fMRI. Pain-evoked CBV-weighted fMRI first revealed that gouty arthritis enhanced CBV increases in the primary somatosensory cortex and thalamus. Examining the TRPV1 expression of these areas confirmed that TRPV1 level was upregulated in the cortical area. Reversing TRPV1 activity by intrathecal administration of AMG9810 reduced pain-related gait abnormality and also induced activation of the thalamocortical pathway. These findings in the brain altogether support the importance of nociceptive signaling augmented by gout. Our study provides the first piece of *in vivo* evidence regarding activated thalamocortical nociceptive signaling through enhancing TRPV1 expression during gout attacks.

The spinothalamocortical tracts are a major pathway for processing nociceptive signals in the brain. Carrying sensory information from the periphery, the spinothalamocortical pathway crosses at the level of spinal cord, first terminating in the thalamus and then in the primary somatosensory cortex. BOLD fMRI is a very popular method of detecting activation of the primary somatosensory cortex in both humans and animals, but its sensitivity in the subcortical area is less satisfactory. In the present study, CBV-weighted fMRI, by improving signal to noise ratio, revealed salient subcortical CBV changes in the thalamus and striatum. While no striatal activity changes were detected throughout the time course in the gout model, TRPV1 blockage did decrease striatal CBV responses (Fig. 6B). Our suspicion is that the striatal CBV change normally tends to reach a ceiling, and cannot be enhanced further by the gouty condition. But once a potent inhibitory event like TRPV1 blockade is present, decreases of CBV in the striatal region are revealed.

Whether TRPV1 is intrinsically expressed in brain areas is a debated issue. Two immunohistochemical studies first confirmed the widespread existence of TRPV1 in the brain, including the cortex, hippocampus, amygdala, striatum, thalamus, hypothalamus, cerebellum, etc.^16^. TRPV1 expression occurs in cell bodies and mostly in post-synaptic dendritic spines^17^. Nevertheless, more recently, genetic modification of the TRPV1 locus in mice revealed only minimal expression of TRPV1 in all tissues of a few discrete brain regions. The restricted expression of TRPV1 in the CNS is conserved across species^18^. Besides the issue of central nervous TRPV1 expression, the induction of TRPV1 functional expression is also controversial. For instance, while TRPV1 knockout mice exhibited altered hippocampal synaptic function^19,20^, no effects of TRPV1 agonists on this altered function have been observed^21,22^. These debates complicate the potential uses of TRPV1-based drugs for treating gout pain. In this study, we adopted a different strategy. Before we used immunohistological and western blotting methods to map the distribution of TRPV1 with and without gouty arthritis, fMRI was employed first to map out the nociceptive pathway in the brain. Therefore, we could focus our assessment of TRPV1 expression levels on areas that exhibited CBV changes in response to electrical stimulation. The findings from this study using fMRI combined with an immunohistochemical/western blotting approach may provide additional insights into the functional role of TRPV1 in the normal brain, as well as the gouty arthritis scenario.

The intrathecal injection route of this study should be contrasted with routes used in previous studies. One previous study verified the significance of TRPV1 by co-injecting TRPV1 antagonist with MSU (1.25 mg/site) intraarticularly^23^. It found that TRPV1 antagonism blocked both inflammatory and pain responses. Since the intraarticular injection mainly acts upon the tissues with MSU deposits, its effects on the resolution of the symptoms was directly due to the dissipation of peripheral events that involve both the inflammatory factors and TRPV1 signaling. In our study, we took a step further by injecting the TRPV1 antagonist intrathecally into the cisterna magna. Such drug administration tends to exert the effects through the cerebrospinal fluid circulating in the ventricular space, and therefore mainly affects the neural tissues around the injection site. This administration was chosen as well because intracerebral injection tends to compromise fMRI signal qualities, which should be avoided whenever possible. Our intrathecal route results are consistent with those obtained using intraarticular injection, except that, interestingly, the wrist swelling was not altered by the intrathecal TRPV1 antagonist. This suggests that, in the case of intraarticular injection, the effects of TRPV1 blockage may involve reducing levels of MSU-related inflammatory factors in the tissue, and therefore this may be a confounding factor in estimating the real significance of TRPV1 in gout pain. In contrast, our intrathecal injection eliminates the contribution from the inflammatory events at the peripheral joint and surrounding soft tissues, and therefore establishes the unique importance of TRPV1 in the nervous system in pain augmentation in gouty arthritis.

Monocrystalline iron oxide nanocompounds (MIONs) are ultrasmall superparamagnetic particles of iron oxide. The particles have a hydrodynamic diameter of 20–30 nm. MIONs have a longer blood half-life and induce a relatively stable signal curve^24^, and therefore are more suitable for use in steady state CBV fMRI^25^. Larger sized superparamagnetic particles of iron oxide such as Endorem, on the other hand, have a shorter half-life and therefore wash out, leading to drift in the background signal. When using SPIO as a contrast agent for CBV fMRI, detrending approaches and pharmacodynamics modeling are required for more accurate quantitative comparison^26,27^.

### Limitations

We can not rule out the possibility that the increase in CBV is also explained by greater abrupt elevation of mean arterial blood pressure (MABP) in response to applied stimuli that are reflected in the activated areas. Indeed, elevated MABP has been considered a confounding variable of fMRI hemodynamic responses^28,29^. This is an issue in nociceptive fMRI: noxious stimuli not only elicit neural activity that cause hemodynamic changes, but also abrupt changes in MABP that can lead to a stimulus-correlated increase in the influx of oxygenated blood from the periphery into the brain vasculature and thus to an increase in cerebral blood flow (CBF)^30^. The BOLD effects of large veins are prone to reflect abrupt elevations in MABP in response to the stimulus. MABP increases can also affect CBV measurements, but it is the least sensitive metric to MABP-induced effects compared to BOLD and CBF.

The 96-hr fMRI response to paw injection of saline seen in the contralateral S1 indicates a CBV decrease. This elusive response could be due to blood steal or neurotransmitter release, but further investigation is needed for clarification.

## Conclusions

Non-steroid anti-inflammatory drugs and colchicine are first-line agents for the acute gout attack^31^, but these drugs are poorly tolerated or contraindicated in some patients. Elucidating the nociceptive signaling pathway of gout may shed light on the key molecules that may be pursued as therapy targets in the future. Our neuroimaging, cellular, and molecular investigations collectively reveal activation of a novel transduction pathway from the periphery to the brain during gout attacks. Our data support that (1) increased TRPV1 expression along the thalamocortical pathway is critical to the development of gout pain, and (2) suppressing central TRPV1 activity is effective in alleviating the pain. Moreover, fMRI could be a very useful tool for potential drug target identification. Besides TRPV1, additional proteins or other TRPV channel proteins may be assessed by similar techniques and thus add more to our understanding of gout pain in the future.

## Materials and Methods

### Gouty arthritis rat model

Male Sprague-Dawley rats (n = 36 as specified in Fig. 1A; 8 weeks old; 300–320 g body weight; National Laboratory Animal Center, Taiwan) were used. All experimental procedures were approved by the Institute of Animal Care and Utilization Committee at Academia Sinica, Taiwan. All methods were performed in accordance with the relevant guidelines and regulations. The gouty arthritis rat model was induced by injection of MSU into the wrist. Briefly, 0.42 g of uric acid (U2625; Sigma-Aldrich, MO, USA) was dissolved in 100 mL of distilled water alkalinized by the addition of 0.1 g of sodium hydroxide (Sigma-Aldrich). After leaving the solution overnight at room temperature, MSU crystals were harvested by decanting off the supernatant solution and washing three times in cold sterile phosphate-buffered saline (PBS). Crystals were then left to dry at room temperature for five days before re-suspension in PBS at a concentration of 24 mg/mL and sterilizing in an autoclave. The rats received injection of 50 μL of 24 mg/mL MSU crystals directly into the left wrist joint cavity and injection of 50 μL of saline directly into the right wrist as the control^32–34^. Overt signs assessment, fMRI, immunohistochemistry, or Western blotting were carried out at 3, 24, 48, and 96 hr after MSU crystal deposition, as indicated in the timeline of Fig. 1A.

### Observation of overt signs in rats with gouty arthritis

The examination was focused on the gait and wrist swelling, two key overt signs in rats with gouty arthritis^35^. Gait is believed to correlate with the pain degrees, and was scored according to the following criteria: 0 = normal gait; 1 = slight limp, the paw of injected forelimb completely touches the floor with closed fingers; 2 = moderate limp, the paw of injected forelimb only briefly touches the floor; 3 = severe limp, three-legged gait (the paw of injected forelimb elevates off the floor when walking)^36^. The rats with three-legged gait at 3 hr after MSU injection received subsequent fMRI assessment and their tissue were handled for immunohistochemistry, or Western blotting. The wrist swelling is a recognized index of inflammation, and it was determined by measuring the thickness of the wrist with a vernier caliper^37^.

### CBV weighted-fMRI evoked by sensory stimulation

CBV-weighted fMRI was performed at the designated time points using a 4.7-T spectrometer (Biospec 47/40, Bruker, Germany) with a 72-mm-volume coil as the radiofrequency transmitter, and a quadrature surface coil as the receiver. Anesthesia was first induced in the animal with 5% isoflurane in oxygen at flow rate of 5 L/min, and then maintained with 〈-chloralose (70 mg/kg) dissolved in heated 0.9% saline and 10% PE glycol administered via an intubated femoral vein. The rat was then fixed in a customized holder to minimize motion artifacts. Throughout the experiment, the animals were allowed to breathe spontaneously. According to the settings used in previously studies^38,39^, steady-state CBV-fMRI was performed using monocrystalline iron oxide nanoparticles (MIONs; 15 mg Fe/kg given intravenously; volume 0.25 ml) as the contrast agent. Once the concentration of MIONs reaches a steady state in the circulation, enhanced neural activity increases regional CBV, and thus the quantity of MIONs, leads to a decreased signal intensity on the fMRI images. Conversely, elevated signal intensities represent a decrease in regional CBV^38,40^.

According to previous studies^3–6^, for each fMRI data set, a time series of 60 images were acquired in the axial plane. The 60 time frames were divided into five phases corresponding to the off, on, off, on, and off status of noxious electrical stimulation, respectively, which were delivered to a left forepaw via a pair of needle electrodes. Gradient-echo images were acquired in the time series with a repetition time of 150 ms, an echo time of 15 ms, a flip angle of 22.5°, a field of view of 2.56 cm by 2.56 cm, a slice thickness of 1.5 mm, an acquisition matrix of 128 × 64 (zero-filled to 128 × 128), and a temporal resolution of 9.6 s. A total of five slices were measured, covering the area from Bregma 1.2 mm to Bregma −4.8 mm. The stimulation intensity was 10 mA, administered by a constant-current stimulator (model 2100, A-M Systems, Carlsborg, WA, USA). This intensity has been shown previously to induce pain^6,41,42^, and unilateral electrical stimulation is known to induce CBV decreases in the bilateral striata with CBV increases in the contralateral primary somatosensory cortex (S1)^38^.

Throughout the experiment the animal’s body temperature was maintained at 37 °C using a warm-water blanket. The average end-tidal CO_2_ concentration was 3.0–3.5%. The baseline partial pressure of CO_2_ (pCO_2_) was 41–43 mmHg. The mean arterial blood pressure before stimulation was 95–105 mmHg, and this was increased during electrical stimulation by 5–10 mmHg.

### Image processing and analysis

Correlation maps were generated by plotting the Pearson correlation coefficient (CC) between the image signals and the off–on–off–on–off electrical stimulation paradigm on a voxel-by-voxel basis using the cross-correlation method^43,44^. The cutoff point for the CC was *r* = ±0.3^4,38,39,45^ because it showed clear signal clusters in anatomical structures with few noisy pixels. Moreover, the signal intensities expressed as percentage changes relative to the baseline were plotted as a time course, with a linear correction for the gradual washout of the MIONs. The percentage changes in signal intensity during the two ON phases out of the 60 image frames were averaged as a measurement for statistical analysis. The spatial extent of the identified CBV signals above the thresholds was also quantified from the CBV correlation maps. Analysis of variance (ANOVA) followed by post-hoc *t*-tests were used to discern statistical differences. The cutoff for statistical significance was set at *p* < 0.05 (n = 6 in each group).

### TRPV1 antagonist into the cisterna magna

AMG9810 (2316; Tocris Bioscience, Bristol, UK), a selective TRPV1 receptor antagonist^46^, was infused through the cisterna magna in the rats^47,48^ (n = 6 with treatment and n = 6 with vehicle). One μg of AMG9810 was dissolved in 15 μL of distilled water containing 20% 2-hydroxypropyl-β-cyclodextrin (H107; Sigma-Aldrich)^49^ and loaded into a 50-cm long PE-8 catheter (NATUME, Tokyo, Japan), one end of which was connected to a 0.5 mL insulin syringe (B. Braun, Melsungen, Germany). After anesthetizing the rat with isoflurane (5% in 1 L/min oxygen flow), the skin of the neck was shaved, and the head was then fixed prone in a stereotaxic frame. The surgical site was exposed by flexing the head 90 degrees to the horizontal and then a sagittal incision of the skin was made inferior to the occiput. Under a dissection microscope, the subcutaneous muscles were separated to uncover the dura mater of the cisterna magna. The open end of the AMG9810 solution-filled catheter was inserted into the cisterna magna and fixed by medical adhesive (Histoacryl; B. Braun) after puncturing the dura mater with a 30 G needle. After the intubation, the rat was carefully moved to the animal holder for the following fMRI experiments. The injection of AMG9810 was performed after the 1^st^ fMRI paradigm of electrical stimulation, and its effects were assessed in the 2^nd^ stimulation paradigm.

### Histology and Immunohistochemistry

After the fMRI experiments and/or the behavioral evaluations, rats were perfused transcardially with saline followed by 4% paraformaldehyde in PBS. The brains and the wrist joints were gently removed, post-fixed in the same fixative for 3 hours, paraffinized, and sectioned at 5 μm. The forepaw plantar skins were also collected, post-fixed, immersed in 30% sucrose, and cryo-sectioned at 50 μm.

Hematoxylin and eosin (H&E) staining of the wrist tissues was performed as follows. The slides were first rehydrated in 100%, 95%, and 80% ethanol followed by dH_2_O. The sections were then immersed in Hematoxylin solution for 8 minutes. After washing in running water, the sections were then immersed in Eosin solution for 30 seconds followed by dehydration in 95% and 100% ethanol. Myeloperoxidase (MPO) staining was performed as follows. After rinsing, the slides were incubated for 45 seconds in a diaminobenzidine solution with 0.5 ml of 1% hydrogen peroxide prepared according to MPO kit instructions (Sigma-Aldrich). After rinsing, the slides were then counterstained, washed, and air dried. Before beginning the immunohistochemistry procedure, paraffin and cryo-embedding compound were removed from the sections by xylene/alcohol gradients and PBS, respectively. In brief, the sections were treated with 0.3% H_2_O_2_ in PBS. For brain tissue, the sections were incubated additionally with antigen retrieval solution (S2368; Dako, Glostrup, Denmark) at 98 °C for 20 minutes. The sections were then incubated first with primary antibody overnight at room temperature (RT), then with biotinylated secondary antibody (1:1000; Jackson ImmunoResearch, PA, USA) for 1 hour at RT. For visualization, the sections were incubated with avidin-biotin-peroxidase complex (Vector Laboratories, CA, USA) for 1 hour at RT, and subsequently treated with PBS containing 0.025% diaminobenzidine (DAB), 1.5% nickel ammonium sulfate, and 0.024% H_2_O_2_ until the desired color was developed. The primary antibodies used in the immunostaining were: anti-TRPV1 (1:1000; sc-12498; Santa Cruz Biotechnology, TX, USA); anti-MPO (1:100; A0398; Dako); anti-COX-2 (1:100; RB-9072-P1; Thermo Fisher Scientific, CA, USA). The negative controls were performed using the same conditions, except that primary antibody was excluded (data not shown).

The sections were photographed with a light microscope (BX51; Olympus, Tokyo, Japan) and the images were analysed with ImageJ (NIH, MD, USA) as reported previously^50^. In the brain sections, TRPV1 signal intensity and TRPV1-expressed area fraction were quantified. Briefly, the relative optical intensity was the optical intensity measured on inverted gray-scale images and normalized to the mean gray-scale value of the control. The area fraction was calculated from the areas with signal intensity greater than the mean gray-scale value of the controls by subtracting one standard deviation and normalizing the result to the total photographed area. All photos were processed by research staff blinded to the experimental conditions.

### Western blot and ELISA

After CO_2_ euthanasia of the rats, brain cortex and thalamus tissues and the forelimb wrists were immediately removed and frozen in liquid nitrogen. The collected tissues were homogenized in lysis/extraction buffer (T-PER; 78510; Thermo Fisher Scientific) containing protease inhibitor cocktail (P8340; Sigma-Aldrich) and then measured with a protein assay kit (Bio-Rad Laboratories, CA, USA) to obtain the protein concentration of each homogenized sample. To evaluate brain TRPV1 level, the extracted proteins were immunoprecipitated with anti-β-tubulin antibody prior to western blot analysis to reduce the background of anti-TRPV1 antibody since TRPV1 is largely bound to β-tubulin^51^. In the western blot, samples with equal amounts of protein were subjected to SDS-PAGE and the separated constituents were transferred to a nitrocellulose membrane by electroblotting. The blots on the membrane were blocked with 5% milk for 1 hour at RT and incubated with primary antibody overnight at 4 °C. The blots were then incubated with horseradish peroxidase-conjugated secondary antibody (1:5000; Jackson ImmunoResearch) for 1 hour at RT, treated with chemiluminescence reagent using an ECL kit (enhanced chemiluminescence; Amersham Biosciences, Buckinghamshire, UK), and visualized by autoradiography. The primary antibodies used were: anti-TRPV1 (1:2000; PC420; Merck Millipore, Darmstadt, Germany); anti-COX-2 (1:1000; Thermo Fisher Scientific); β-tubulin (1:2000; MCA2703; Bio-Rad Laboratories); β-actin (1:4000; sc-47778; Santa Cruz Biotechnology). Quantitative densitometric analysis of the bands was conducted with ImageJ according to instructions by NIH. COX-2 level in the wrists was normalized to β-actin level while TRPV1 level (after immunoprecipitation) was normalized to β-tubulin level and then to control level^52^.

The concentrations of PGE2 and bradykinin in the wrist homogenates were further analysed by ELISA kits (PGE2: KGE004B, R&D Systems, MN, USA; bradykinin: EK-009-01, Phoenix Pharmaceuticals, CA, USA) according to the manufacturer’s instructions and expressed as ng per μg wet tissue.

## Data Availability

The datasets generated during and/or analysed during the current study are available from the corresponding author on reasonable request.
